# Oligo kernels for datamining on biological sequences: a case study on prokaryotic translation initiation sites

**DOI:** 10.1186/1471-2105-5-169

**Published:** 2004-10-28

**Authors:** Peter Meinicke, Maike Tech, Burkhard Morgenstern, Rainer Merkl

**Affiliations:** 1 Abteilung Bioinformatik, Institut für Mikrobiologie und Genetik, Georg-August-Universität Göttingen, Goldschmidtstr. 1, 37077 Göttingen, Germany; 2 lnstitut für Biophysik and physikalische Biochemie, Universität Regensburg, Universitätsstr. 31, 93040 Regensburg, Germany

## Abstract

**Background:**

Kernel-based learning algorithms are among the most advanced machine learning methods and have been successfully applied to a variety of sequence classification tasks within the field of bioinformatics. Conventional kernels utilized so far do not provide an easy interpretation of the learnt representations in terms of positional and compositional variability of the underlying biological signals.

**Results:**

We propose a kernel-based approach to datamining on biological sequences. With our method it is possible to model and analyze positional variability of oligomers of any length in a natural way. On one hand this is achieved by mapping the sequences to an intuitive but high-dimensional feature space, well-suited for interpretation of the learnt models. On the other hand, by means of the kernel trick we can provide a general learning algorithm for that high-dimensional representation because all required statistics can be computed without performing an explicit feature space mapping of the sequences. By introducing a kernel parameter that controls the *degree *of position-dependency, our feature space representation can be tailored to the characteristics of the biological problem at hand. A regularized learning scheme enables application even to biological problems for which only small sets of example sequences are available. Our approach includes a visualization method for transparent representation of characteristic sequence features. Thereby importance of features can be measured in terms of discriminative strength with respect to classification of the underlying sequences. To demonstrate and validate our concept on a biochemically well-defined case, we analyze *E. coli *translation initiation sites in order to show that we can find biologically relevant signals. For that case, our results clearly show that the Shine-Dalgarno sequence is the most important signal upstream a start codon. The variability in position and composition we found for that signal is in accordance with previous biological knowledge. We also find evidence for signals downstream of the start codon, previously introduced as transcriptional enhancers. These signals are mainly characterized by occurrences of adenine in a region of about 4 nucleotides next to the start codon.

**Conclusions:**

We showed that the oligo kernel can provide a valuable tool for the analysis of relevant signals in biological sequences. In the case of translation initiation sites we could clearly deduce the most discriminative motifs and their positional variation from example sequences. Attractive features of our approach are its flexibility with respect to oligomer length and position conservation. By means of these two parameters oligo kernels can easily be adapted to different biological problems.

## Background

During the last years, a large number of machine learning approaches have been developed to analyze and annotate genomic sequence data. Different concepts of statistical pattern analysis and modelling are successfully used for this purpose. Sophisticated methods like Hidden Markov Models [1], neural networks [2] or support vector machines (SVM) [3] became indispensable concepts and are routinely utilized in computational biology. Kernel-based learning algorithms, like SVM, are among the most advanced machine learning methods. As compared with probabilistic (Markov) models and classical neural networks, SVM provide a well-understood regularization [4] mechanism which makes learning from few examples in high-dimensional feature spaces possible. In that way, SVM and related methods can effectively cope with the "curse of dimensionality", which has been difficult for the more traditional tools in machine learning. Together with the kernel trick which provides a technical basis for learning in arbitrarily high-dimensional spaces, the principled approach to regularization has been the foundation for a large variety of successful applications of SVMs to realworld pattern-classification tasks [3]. However, a certain drawback of kernel methods is that, from a user's point of view, they usually behave like black boxes. Once the training phase is done, it is not easy to identify the features which actually determine the quality of the classification. This fact complicates the design of such systems and an interpretation of the learnt representation. Therefore additional feature selection procedures have been proposed to cope with that inherent shortcoming of traditional kernel methods. In that way, Degroeve *et al*. [5] proposed to combine SVM based on traditional kernels with techniques for feature selection in order to localize mononucleotide occurrences relevant for the prediction of splice sites.

Herein, we introduce *oligo kernels *which provide a novel approach to datamining on biological sequences, based on the powerful concept of kernel feature spaces [6]. Our approach has two main advantages compared to previous applications of kernel methods to biological sequence analysis: (*a*) the existing methods provide either position-dependent representations based on mononucleotide occurrences [5,7] or they consider general *K*-mer occurrences restricted to a completely position-independent representation [8,9]. By contrast, with our method it is possible to adjust the required level of position-dependency to any degree for oligomers of any length. This convenient feature, in turn, facilitates the modelling of positional and compositional variability of complex biological signals. On the other hand, as we shall show in the next section, traditional monomer-based representations and position-independent *K*-mer representations may also be realized by the oligo kernel. (*b*) Furthermore, our method provides an intuitive visualization approach to present relevant oligomers and their positional variability to the user. For that purpose a suitable measure of discriminative power can be utilized to score different motifs according to their relevance for classification. Oligo kernels can therefore be applied to infer characteristic sequence features which, in turn, can be used to identify functionally important signals. This property qualifies the oligo kernel as a useful datamining tool for the analysis of biological sequences.

To demonstrate the usefulness of our approach, we applied oligo kernels to analyze translational initiation sites (TIS) for *Escherichia coli *K-12 because a reliable set of biochemically verified sites is available for that case. Prediction of TIS is not satisfactorily solved for prokaryotic genes [10]. For bacteria, there are a number of gene-finding tools that reliably predict the location of genes in a genome under study. Essentially, these methods work by looking for open reading frames of a certain statistically significant minimal length. But while it is obvious how to identify the *end *position of a predicted gene, it is by no means trivial to determine the corresponding *start *position as start codons are not unique; they are also used to code for amino acids *inside *genes. Systematic studies have shown that existing gene-prediction programs perform poorly when it comes to predicting the correct TIS [11,12]. Consequently, many start positions are incorrectly annotated in databases and, due to the concepts used for gene annotation, these errors tend to be propagated to newly annotated genomes.

Although our method is based on classification of sequences we do not intend to provide a TIS prediction tool in this paper. On one hand the method provides a general tool for identification and characterization of signals in biological sequences which is not restricted to TIS sequences. On the other hand our datamining approach may be viewed as a prior step for constructing TIS predictors. By means of an easy visual interpretation of the inferred model it can provide new insights which in turn can steer the construction of efficient predictors. We expect that this feature will in particular be useful for analysis of prokaryotic genomes for which only small sets of experimentally verified TIS exist.

### Oligo kernels

Kernel-based learning [6] provides a powerful framework for many kinds of pattern analysis tasks usually encountered in statistical evaluation of experimental data. Given an input space 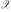 a kernel is simply a function 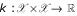. It implicitly applies a usually nonlinear transformation 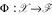 to elements **x**, **x' **in input space 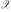 and then computes the inner ("dot") product within a resulting feature space 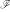:

*k*(**x**, **x'**) = **Φ**(**x**)·**Φ**(**x'**).     (1)

A kernel can be viewed as a similarity measure for the input space objects **x **and **x' **which is defined as an inner product of the feature space objects **Φ**(**x**) and **Φ**(**x'**). The concept of kernel-induced feature spaces may first be motivated by the objective to provide an adequate representation for the data, which is more suitable for the classification task at hand than the original input space 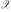. Second, the so-called *kernel trick *suggests to perform learning in that feature space without any explicit computation of the mapping **Φ**, just using inner products of the feature space representatives. The inner products in turn may be computed by some realization of the above kernel function (1) in a hopefully efficient way. That trick may prove useful in situations where the feature space objects are rather high-dimensional and computation of **Φ **is costly. As will be shown below, these objects may even be functions, i.e. objects of infinite dimensionality.

For construction of a linear classifier in feature space the discriminant for the two-class problem requires a feature space weight vector **w **∈ 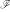 and an scalar offset *b*. With an explicit feature space mapping of the input elements the *primal *form of the discriminant is

*f*(**x**) = **w**·**Φ**(**x**) + *b*.     (2)

The sign of that discriminant can be used to assign **x **to one of the two classes, i.e. to realize a binary classifier. By means of the kernel trick the above primal form can be replaced by its *dual *representation, which does not require access to any feature space elements. The basis for constructing a kernel classifier is a training set of *n *labelled input space examples **x**_1_, **x**_2_,...,**x**_*n*_. Using these examples, we can construct a linear discriminant in feature space without explicit computation of the feature space representatives. Instead we only compute inner products according to the above kernel function weighted by parameters *α*_*i *_in order to get the dual form of the discriminant:


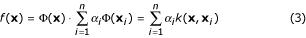


where we assumed *b *= 0 for convenience. The weights *α*_*i *_determine how much individual training examples contribute to the discriminant. Optimal values have to be determined by some suitable learning algorithm which will be outlined below.

Unlike most SVM approaches which just propose a kernel and do not care about the primal form of the discriminant, i.e. about interpretability, we here first propose a suitable primal representation and then apply the kernel trick in order to provide a general learning scheme for that discriminant. By means of the primal/dual-concept of *oligo functions *and *oligo kernels *we achieve both: learnability *and *interpretability of the proposed model. For a suitable realization of the primal form, the idea is to represent oligomer (*k*-mer) occurrences in terms of smooth functions which preserve positional information about oligomer locations to an adjustable extent. In order to model positional variability of the underlying biological signals we introduce some measure of *positional uncertainty*. The degree of positional uncertainty is controlled by some smoothing parameter *σ *which allows the model to be selected from a continuous space of candidates between the two extreme cases of completely position-dependent and position invariant models, respectively. An important advantage of the oligo kernel over previous position-dependent kernels is that, besides the dual representation of the feature space discriminant which is suitable for learning, it also provides a primal representation which is suitable for interpretation of the learnt discriminant. Therefore, classifiers based on oligo kernels not only provide a method for predicting signals in sequences but they may also help to identify relevant *K*-mers of any length and to analyze their positional variability. The two kinds of representations are detailed in the following.

### Primal representation: oligo functions

**Figure 1:**
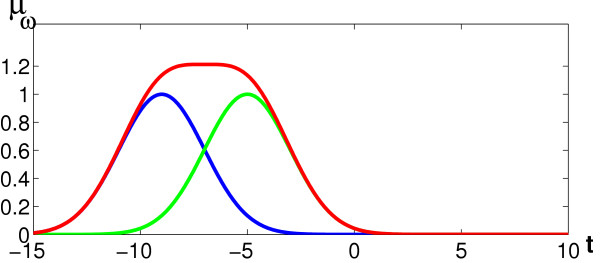
**Illustration of positional uncertainty. **Figure 1 illustrates how *positional uncertainty *is represented by the proposed oligo functions. In the example, we have an occurrence of a certain oligomer at position -5 (green curve) and -9 (blue curve). The degree of uncertainty depends on the variance *σ*^2 ^of the Gaussian bumps centered on these positions. Adding the two Gaussians, the smoothness of the resulting oligo function (red curve) increases with increasing *σ *and the assignment of the oligomer to certain positions becomes more fuzzy.

For the primal representation of the feature space discriminant (2) we introduce the concept of *oligo functions *which encode occurrences of oligomers in sequences with an adjustable degree of positional uncertainty. For that purpose, occurrences are not represented by exact ("hard") assignment of the oligomers to their observed positions, but rather by some kind of "fuzzy" assignment according to the assumed positional uncertainty. Therefore oligo functions can be viewed as fuzzy membership functions [13], up to an arbitrary scaling, which in the case of fuzzy membership functions restricts the function values to the range [0,1]. In that way oligo functions assign *K*-mer occurrences to positions in a "soft" manner. For a convenient realization we choose these functions to be mixtures of Gaussians with the variance *σ*^2 ^of the Gaussians controlling the degree of positional uncertainty. Thus, for an alphabet 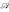 and a sequence **s **which contains *K*-mer *ω *∈ 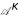 at positions *S*_*ω *_= {*p*_1_, *p*_2_,...} we obtain the oligo function


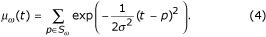


with continuous position variable *t *which does not need to be restricted to a discrete domain so far. The smoothing parameter *σ *adjusts the width of the Gaussians which are centered on the observed oligomer positions and therefore it determines the degree of position-dependency of the function-based feature space representation. While small values for *σ *imply peaky functions, large values imply flat functions with the limiting case *σ *→ ∞ preserving no positional information at all. The effect of positional uncertainty is shown in figure 1 where the Gaussian bumps of two distinct oligomer occurrences result in a single bump of the corresponding oligo function. Consequently, for the sequence **s **the occurrences of all *K*-mers contained in 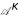 = {*ω*_1_, *ω*_2_,...,*ω*_*m*_} can be represented by a vector of *m *oligo functions which yields the final feature space representation of that sequence:


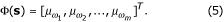


Note, that the feature space objects are vector-valued functions, which can be stressed using the following notation:

*φ*_**s**_(*t*) = [*μ*_1_(*t*), *μ*_2_(*t*),..., *μ*_*m*_(*t*)]^*T *^    (6)

where we used the shortcut 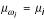. Thus, the feature space representatives are curves in the *m*-dimensional space of all *K*-mers.

If we discretize the oligo functions according to the actual number of sequence positions, we would be able to assemble feature vectors of finite dimensionality, just stacking the vectors *φ*_**s**_(*t*_*i*_) of function values for all positions *t*_*i *_considered. Then, usual vector-based learning algorithms could be applied in order to infer relevant sequence characteristics. Unfortunately, this approach would only be feasible for short oligomers, i.e. for small *K*, because already for DNA sequences with *l *positions the number of required vector dimensions is *l *× 4^*k *^while for protein sequences it is *l *× 20^*K*^. In our application we used *l *= 200 positions and therefore we would have got nearly a million dimensions for representation of hexamer occurrences, which is prohibitive for usual vector-based learning schemes.

#### Visualization

While oligo functions in general cannot be used directly for learning of a feature space discriminant they are well-suited for the interpretation of a learnt discriminant. The latter fact is an important prerequisite for our datamining approach which provides an intuitive insight of the kernel-based sequence model. Considering the primal form of the feature space discriminant, the weight vector becomes a vector-valued function arising from a linear combination of the feature space representations of the sequences. With the learnt parameters *α*_*i *_we can construct the vector-valued weight function of the discriminant:


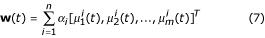


which is a curve in the *m*-dimensional space of oligomers. For each of the *m *components we have a linear combination of oligo functions where the weights *α*_*i *_determine the contribution from each of the *n *training sequences. Due to its primal representation based on oligo functions, an oligo kernel classifier provides an intuitive insight into its discriminant because high positive (negative) values of the weight function contribute to prediction of the positive (negative) class. So the user can easily inspect the learnt representation which in turn allows him to draw conclusions about the relevance of certain oligomers and their locations in terms of their discriminative power.

In order to facilitate the interpretation of the discriminant, one may restrict the analysis to the most discriminative oligomers. This can be done by ranking the component weight functions of **w**(*t*) = [*w*_1_(*t*), *w*_2_(*t*),..., *w*_*m*_(*t*)]^*T*^ according to their *L*_2_-norm


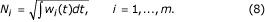


Because higher norms indicate a more important role in discrimination, selection of the corresponding weight functions helps to keep the focus on the relevant oligomers. A similar criterion has also been suggested for the position-independent spectrum kernel in order to identify discriminative motifs which can be used to distinguish real exons from pseudo exons [14].

Because the feature space weight vector can be represented as a vector of functions, a suitable *visualization *of these discriminative weight functions provides complete access to the information which has been extracted from the data. For an overview, all discriminative weight functions *w*_*i *_may be discretized and stored in a matrix which may be visualized as a bitmap image using grey values or color to encode the function values. Using *l *discrete sequence positions *t*_*i*_, the *m *× *l *image matrix can easily be obtained as

**W **= [**w**(*t*_1_),**w**(*t*_2_),...,**w**(*t*_*l*_)].     (9)

For longer oligomers only a subset of weight functions may be used, which can be selected according to the above *L*_2_-norm. For a closer look on the role of single oligomers the corresponding weight functions can be visualized as usual 2D function plots. Examples for both kinds of visualization will be shown in the section on results.

### Dual representation: oligo kernels

In the previous section we have derived a feature space representation for our biological sequences, where a certain sequence **s**_*i *_from our dataset is represented by a vector of oligo functions, i.e. a curve 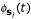 in the *m*-dimensional space of all *K*-mers. While this feature space representation is well-suited for interpretation of the discriminant, it is impractical for learning the discriminant from sequence data. For that purpose we shall utilize the dual form of the discriminant, based on *oligo kernels*. By means of the kernel trick the dual representation is well-suited for training and any kernel-based learning algorithm [6] may be utilized. With the shortcut 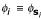 an inner product of two sequence representations *φ*_*i*_, *φ*_*j *_can be defined as


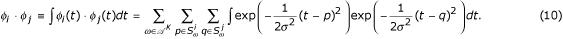


Note that the second integral is a function of the distance *d *= |*p *- *q*| between two oligo positions *p *and *q*. This function *I*(*d*) is equivalent to the convolution of two Gaussians with equal variance. Up to some constant factor, the convolution of two Gaussians is a Gaussian whose variance is the sum of the original variances. Therefore the integral can be calculated according to


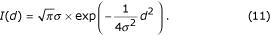


Replacing the second integral in (10), the oligo kernel, i.e. the inner product of two feature space representatives, can be computed according to


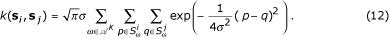


From the above definition of the oligo kernel, it is easy to see the effect of the smoothing parameter *σ*. For the limiting case *σ *→ 0 with no positional uncertainty, only oligomers which occur at the same positions in both sequences contribute to the sum. In general it would not be appropriate to represent oligomer occurrences without positional uncertainty, which would imply zero similarity between two sequences if no *K*-mer appears at *exactly *the same position in both sequences. Regarding the other extreme *σ *→ ∞ with maximum positional uncertainty, position-dependency of the kernel completely vanishes: all terms of oligomers, occurring in both sequences, contribute equally to the sum, regardless of their distance. Together with the normalization which is introduced below, in the latter position-independent case the oligo kernel becomes identical to the so-called spectrum kernel [8] which has been proposed for position-independent representation of sequences.

Regarding computational complexity of the oligo kernel, for two sequences of length *l*_1 _and *l*_2_, respectively, the above oligo kernel (12) can be computed by evaluation of at most *l*_1 _× *l*_2 _exponential functions. For a speed-up of the computations these evaluations may be realized by fast table lookups. Fortunately, the maximum number of *l*_1 _× *l*_2 _evaluations is hardly reached in practice, because we only have to compute terms of (12) for oligomers occurring in *both *sequences. Only in cases where oligomers often occur at many positions in both sequences the complexity is rising towards *O*(*l*_1 _× *l*_2_). These cases become more unlikely for longer oligomers and therefore, with an efficient implementation, the computational cost rapidly decreases with increasing oligomer length *K*.

#### Normalization

According to the above derivation, the feature space representations may have different norms. Here, for the representation of biological sequences, the norm of a feature space object roughly corresponds to the absolute count of the oligomer occurrences. In order to improve comparability between sequences of different length, the feature vectors should be normalized to unit *L*_2_-norm. Therefore we compute the normalized oligo kernel according to


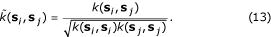


#### Training

The basis for learning a kernel classifier is a training set of *n *labelled example sequences 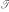 = {(**s**_1_,*y*_1_), (**s**_2_,*y*_2_),...,(**s**_*n*_,*y*_*n*_)} with *y*_*i *_= 1 or *y*_*i *_= -1 for positive and negative examples, respectively. Because we want to propose a general learning scheme which is even applicable to problems with small data sets, a *regularized *learning scheme is essential in order to prevent overfitting. Many regularized learning schemes are available, among which soft-margin SVMs [15] are usually best-known. For convenience, we here use regularized least squares classifiers [16], which have been shown to provide equivalent performance on many classification problems, as compared with SVMs. Implementation of these kernel classifiers is very simple because training can be achieved by solving a system of *n *linear equations, as shall be outlined below. Given the set 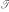 containing *n *labelled sequences, with labels *y*_*i *_∈ {-1,1} contained as components in *n*-vector **y**, we shall now train a kernel classifier based on discriminant (3) using the proposed oligo kernel. With the kernel matrix 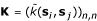 which contains all possible inner products of the training set examples in feature space, we realize a classifier by minimizing the *λ*-penalized prediction error


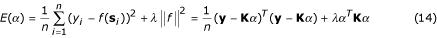


with respect to parameter vector *α *= [*α*_1_,...,*α*_*n*_]^*T*^. By means of the regularization parameter *λ *> 0 the penalty controls the norm of the feature space discriminant in order to avoid overfitting. Bounding the norm of the discriminant restricts the learning algorithm to put higher weights only on "effective" features which are important for classification. In that way learning is forced to focus on that task-specific information which can actually be drawn from the data. Choosing the parameter vector *α *to yield a minimum of the above error, an optimal realization can be found by solving the following system of linear equations:

(**K **+ *λn***I**)*α *= **y **    (15)

where **I **is the *n *× *n *identity matrix. Because solving the above system in general is of complexity *O*(*n*^3^) the computational cost of the learning algorithm is rapidly increasing for an increasing training set. In practice we observed that effective routines for matrix inversion enable training with a few thousands of examples. However, on one hand this behavior is a general shortcoming of kernel methods and recently several approximations have been suggested, which can effectively decrease the computational cost [17,18]. With these approximations training with a few 10000 of examples becomes feasible. On the other hand, for our case study on prokaryotic TIS we encountered data sets with at most 3500 exemplary TIS sequences which can even be handled with the above learning scheme.

## Results

### Datasets

In order to create a reliable dataset we utilized *E. coli *genes from the EcoGene database [19] and considered only those entries with biochemically verified N-terminus. For training, validation and testing of classifiers for both the positive and negative examples we chose the sequences according to large windows of 200 nucleotides (nt) length around the candidate start codon, in order to encounter the risk of missing relevant information. The positive examples were 722 sequences covering a range of 100 nt upstream to 99 nt downstream of the annotated TIS. For the negative examples we extracted sequences centered around a codon from the set {ATG, GTG, TTG} and accepted sequences, if the codon was in-frame with one of the appropriate start sites used as positive case, if its distance was < 60 nt and if no in-frame stop codon occurred in between. The rationale for this approach comes from our analysis of predictions for the *E. coli *genome of those tools integrated into YACOP [12]: We found that nearly two-thirds of the predictions inconsistent with the annotated TIS were located within a distance less than 50 nt to its respective start codon (data not shown). Therefore we concluded that these false sites are the most difficult candidates for TIS discrimination. We finally obtained a set of 854 negative examples with 576 of them being located downstream and 278 upstream of a TIS.

## 

### Prediction performance

To analyze the TIS sequences by means of the proposed oligo kernel, first of all we tested the predictive power of the feature space representation as it depends on different oligomer lengths. In order to compare their discrimination performance, we trained classifiers using oligo kernels according to *K*-mers of length *K *= 1,...,6. For a reliable estimate of the prediction error we performed 50 runs for each oligomer length where each run comprises training, validation for adjusting the hyperparameters *σ *and *λ *and final testing on data which have not been used to adjust the parameters of the classifier. The latter was done on a test set which contained one third of the data. From the remaining data two thirds were used for training and one third for validation of the classifiers with respect to hyperparameter values. Thus, for each run the data were randomly partitioned into training, validation and test sets of size 631, 378 and 567, respectively. For validation we varied the smoothing parameter *σ *∈ {0.5, 0.75, 1, 1.5, 2} and the regularization parameter *λ *∈ {0.1·0.9^*i*^|*i *= 0, 1,..., 100} in order to minimize classification error on the validation set. With the optimal hyperparameter values we then trained a classifier on the union of training and validation set. Finally that classifier based on optimal hyperparameters was evaluated on the test set to yield the final classification error. The mean test error over the 50 runs together with the corresponding standard deviation is shown in table 1. Additionally, table 1 also shows the mean optimal *σ *over the 50 runs. The table shows that the lowest error rate of 8.9 percent has been achieved for the 3-mer kernel with a mean value 1.25 of the hyperparameter *σ *which models the positional variability of the oligomers. This result indicates that the best representation does not necessarily require the lowest positional variability. Note that the mean error is *monotonically *increasing for decreasing oligomer lengths below and for increasing lengths above the best length 3. Therefore we did not try to model the occurrences of *K*-mers for *K *> 6. To investigate the effect of an enlarged data set, we chose additional TIS sequences according to GenBank annotations to yield a data set with about four times the number of examples of our original EcoGene-based set. We included all non-hypothetical coding sequences from the *E. coli *(U00096) dataset, including those from the EcoGene dataset, as positive examples. The negative examples were generated in the same way for the EcoGene-based set. So we obtained an enlarged set of 2980 positive and 3968 negative examples. The enlarged set was randomly partitioned, as described above, with the same proportions of training, validation and test sets. The average performance over 20 runs with different partitions is shown in table 2. Unfortunately, the increased data set size resulted in a worse performance for all oligomer lengths, although an improvement would have been expected. Therefore we decided not to use the enlarged data set for further analysis because we could not exclude the possibility of a large number of erroneous annotations for TIS which had not been verified experimentally.

### Visualization

**Figure 2:**
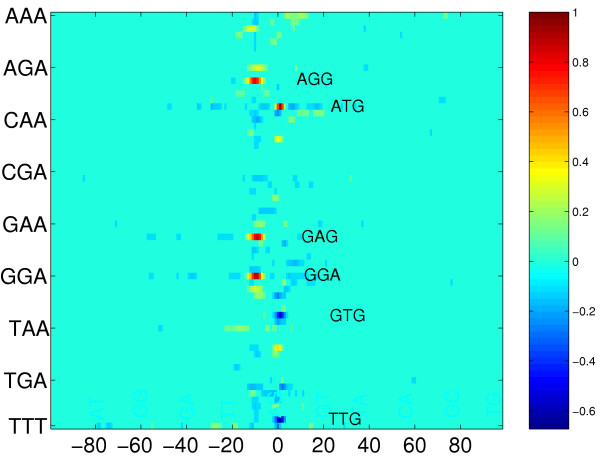
**Image matrix of discriminative weight functions derived from trained classifiers based on the trimer kernel. **Each of the 64 lines shows the values of one trimer-specific weight function obtained from an average over 50 runs (see text). Each of the 200 columns corresponds to a certain position with 0 indicating the position of the start codon. The function values are visualized by the color of the corresponding matrix elements. The complete matrix of function values has been scaled to yield a unit maximum which is located at the ATG line at position 0. For noise reduction all matrix elements with an absolute value below 0.1 have been zeroed. In general, maxima and minima indicate discriminative features which contribute to the prediction of positive (true) and negative (false) TIS, respectively. Note that the region of discriminative features is rather small and mainly concentrated around the start codon on the left (upstream) side of the image.

**Figure 3:**
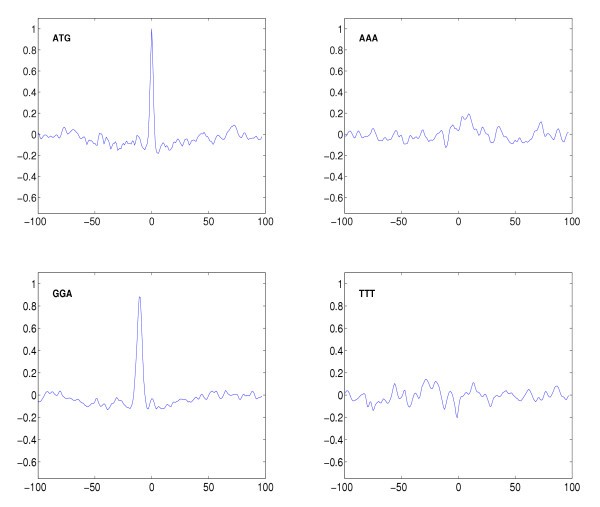
**Exemplary weight functions derived from trained classifiers based on trimer kernel. **Shown are discriminative weight functions for ATG (the most frequent start codon) GGA (having its highest peak in Shine-Dalgarno region), AAA (showing a weak maximum downstream of the start codon) and TTT (with higher values in a region ≈ 20 nt upstream the start codon); function values are plotted versus position. All values are normalized, i.e. they are relative values with respect to a unit global maximum over all functions. The complete set of weight functions for *K *= 3 can be found on our web page [20].

For interpretation of the learnt TIS models, we applied the visualization techniques which have been described above. To visualize the feature space discriminant we first generated an overview bitmap image of the matrix **W **in equation (9) which contains the discretized functions of the primal form weight curve (7) as rows. The image contains the discriminative functions as horizontal lines, with one line for each oligomer. The color of a pixel indicates the level of the corresponding function at each integer position within a window of length 200 nt. Figure 2 shows a 64 × 200 pixel image which was obtained from an average over all optimal **W**-matrices from the above 50 runs using the trimer kernel. The complete matrix of function values is scaled to yield a unit maximum which is attained by the ATG-function at position 0. In addition, for noise reduction all matrix elements with an absolute value below 0.1 are set to zero. In figure 3 four exemplary weight functions for trimers ATG, GGA, AAA and TTT are depicted as 2D-plots showing more detailed information. The complete set of discriminative functions for *K = *3 can be found on the web page [20].

### Oligomer ranking

**Figure 4:**
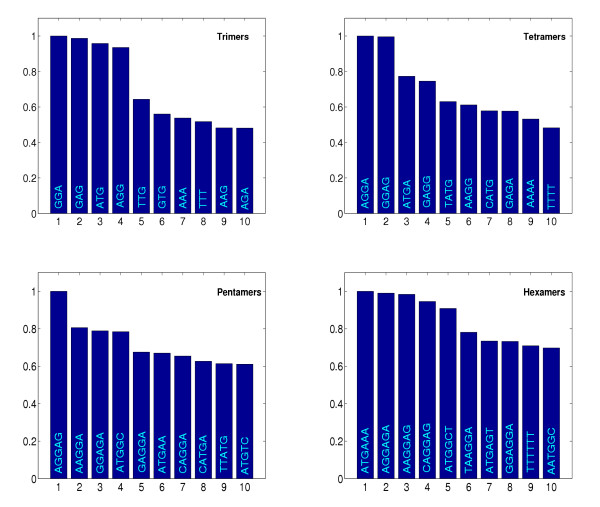
**Oligomer Ranking. **Figure 4 shows the ten most important oligomers for discrimination based on the trimer, tetramer, pentamer and hexamer kernels. The bars show the relative norm of the oligomer-specific weight functions (see text), i.e. their relevance for classification. All values have been scaled to a unit maximum norm of the most discriminative oligomer.

In the following we use the terms monomer, dimer etc. instead of the more specific terms mononucletide dinucleotide etc. In order to identify the most important *K*-mers for the kernel-based TIS prediction, we computed the *L*_2_-norm in equation (8) for all oligomer-specific weight functions of the learnt discriminants. The ten most discriminative *K*-mers are identified using the average norm over the above 50 runs. The resulting rankings for *K = *3,...,6 are depicted as bargraphs in figure 4. The height of the bars is proportional to the average norm of the corresponding *K*-mer weight function and has been scaled to yield a unit maximum height for each *K*. For the monomer kernel we found that A is most discriminative followed by G, T, C in decreasing order of the norm. For dimer occurrences the most discriminative oligomer is GG followed by GA, AG, AT, TT and AA, again in decreasing order. For the longer *K*-mers depicted in figure 4, from the bargraphs one can identify two major groups of motifs which are most prominent: on one hand the start codon itself is an important signal for TIS prediction. Therefore some motifs which contain ATG are associated with high norms and high positive values of the corresponding weight functions at position 0. This can be observed best for the discriminative weight function of trimer ATG itself (see figure 2 and 3).

From these motifs and the associated weight functions we can conclude that C or T at position -1 and A at position 3 seem to be characteristic for TIS as they show high positive peaks at the corresponding positions (see also the above web page). From the pentamer and hexamer occurrences one can deduce a preference for AAA or GCT as a second codon. Start codons GTG and TTG are not characteristic for TIS and therefore the discriminative weight functions of these trimers show high negative peaks at position 0 which can be seen from the overview matrix image of figure 2.

On the other hand oligomers contained in AAGGAGA or GAGGAGA have high rank. The corresponding discriminative functions usually put high positive weights on regions located about 10 nt upstream the start codon (see GGA in figure 2 and 3 or GAG and AGG in figure 2). Obviously, these weight functions utilize the presence of a Shine-Dalgarno sequence for discrimination.

Besides the two prominent groups of motifs related to start codon and Shine-Dalgarno sequence, interestingly, poly-A and poly-T motifs seem to be discriminative, too. As it can be seen from the discriminative functions for AAA and TTT in figure 3, poly-A motifs mainly occur downstream next to the start codon while poly-T occurrences seem to be characteristic in a region ≈ 20 nt upstream.

The corresponding discriminative functions for the ten most important oligomers for lengths 1,...,6 can be found on the before mentioned web page.

### Performance comparison

In order to compare our approach with standard methods for modelling of sequence sites, we utilized inhomogeneous, i.e. position-dependent, Markov models which are widely used for the estimation of positional weight matrices. The probabilistic models can easily be estimated from the data and do not require any hyperparameter optimization. For evaluation we utilized one model for each of the two classes (positive/negative) and assigned a sequence to that class with highest probability of the corresponding model. In the case of zero probabilities for both models, as a tie-breaking rule we assigned the sequence to that model with the smallest number of zero probability positions. Classification was considered correct if the model with the smaller number was associated with the right class. For training we estimated the position-specific oligomer probabilities of the model using two thirds of the data while testing was performed on the remaining third. Prediction performance on the test set was again averaged over 50 runs with different random partitions of the data. For comparison with a position-independent kernel we utilized the spectrum kernel (SK) which can be obtained as a special case of the oligo kernel for *σ *→ ∞. Therefore the spectrum kernel was evaluated in the same way as the above oligo kernels. The methods were compared with the best previous oligo kernel, i.e. the trimer kernel (OK_3_), and with a *combined *oligo kernel (OK_1...6_) which incorporates all *K*-mers for *K *= 1,...,6, simply by adding the six different kernels. With respect to the primal representation of the discriminant, adding the kernels means to stack the vectors of oligomer-specific weight functions for different *K*-mer lengths. Obviously this implies an augmented feature space which might combine the advantages of representations based on short *and *long oligomers. For each of the six added kernels we chose the smoothing parameter according to the median of the optimal values obtained from the 50 previous runs with single oligo kernels which resulted in values [0.5, 0.5, 1.0, 1.0, 1.0, 1.0] for the length-specific smoothing parameters [*σ*_1_,...,*σ*_6_].

As can be seen from table 3 the position-dependent oligo kernels yield the best performance, while the best position-independent spectrum kernel with *K*-mer length 2 failed to discriminate TIS correctly for nearly half of the data. With an average error rate of 44.6%, performance was not much better than classification by chance, indicating the importance of position information for TIS prediction. With the combined kernel OK_1...6 _our method could successfully exploit the combination of different *K*-mer representations and slightly improved the performance of the trimer kernel. In addition, table 3 shows that 0th and 1st order Markov models, based on monomer (MM_1_) and dimer (MM_2_) occurrences, respectively, performed equally well. We found that for the data at hand the 0th order Markov model could not be improved using higher order Markov models. Using Markov models above order 1 the performance even broke down, with a resulting error of ≈ 30%. Therefore in our case the Markov model based representations do not provide an adequate tool for analyzing occurrences of oligomers above length 2. As shown in table 3 even for dimers the oligo kernel provides better discrimination than the 1st order Markov model and therefore it may be preferred over the more simple model. Considering the occurrences of monomers the 0th order Markov model performs slightly better than the corresponding oligo kernel with *K *= 1. While theoretically the oligo kernel should not be worse, in practice it is difficult to find the optimal hyperparameters for best discrimination. Performing a two-dimensional grid search for smoothing parameter *σ *and regularization parameter *λ*, it cannot be expected to find the exact global optimum. On the other hand, for monomer occurrences there seems to be less positional uncertainty, so that the smoothing parameter is not likely to improve the representation anyway. Therefore the oligo kernel does not seem to be the best tool for pure analysis of monomer occurrences.

## Discussion

We have introduced a novel concept of datamining on biological sequences and exemplified its application on the analysis of prokaryotic translational initiation sites.

The interpretation of our results makes clear that the most pronounced signal indicating a TIS besides the start codon is the Shine-Dalgarno region [21]. With our approach we found oligomers contained in AAGGAGA or GAGGAGA to be most discriminative. The corresponding discriminative functions indicate that for characteristic TIS these oligomers are located ≈ 10 nt upstream the start codon. For trimers this can be seen in figure 2 and 3. These results correspond to known findings both with regard to composition and to localization [21,22] and confirm the validity of our approach. The variation in spacing between the start codon and the Shine-Dalgarno region determined previously [21], correlates well with the wider peak of the discriminative functions (compare GGA with ATG peak in figure 3). In addition we observed some evidence for a downstream box, which was previously identified as an additional element modulating the expression level [23-25]. The evidence is a weak positive maximum of the discriminative function for AAA (see figure 3) downstream the TIS. The ranking of discriminative hexamer functions (see figure 4) also shows a preference for the codon AAA immediately following the start codon, as proposed in [25]. In addition also GCT seems to be a characteristic second codon as implied by pentamer and hexamer rankings in figure 4.

The analysis of individual oligo functions (see figure 3) makes it possible to identify subtle signals: The slight decay observed in a region ± 40 nt around position 0 in the plots of the discriminative weight functions for ATG and GGA is due to our selection of negative examples (see Datasets). The weak local minima of these functions seen upstream and downstream of position 0 are simply caused by shifted ATG and GGA sites of positive examples occurring also in our negative examples. The positive signal for TAT and CAT at position -1 (see figure 2) is due to the prominence of the oligomer ATG at position 0, but also indicates a preference for T and C at -1. This is also indicated by the tetramer ranking in figure 4 which identifies TATG and CATG as discriminative oligomers.

Comparing our approach with other methods we found that position-dependency is crucial for an appropriate TIS representation. The position-independent spectrum kernel showed a bad discrimination performance which does not qualify that method for prediction or for analysis of TIS sequences. On the other hand the 0th order Markov model showed a competitive performance as compared with the monomer-based oligo kernel. Thus, for the analysis of monomer occurrences the oligo kernel seems to be "oversized" and we cannot recommend its application for merely *that *purpose. However, we argue that most biological signals are not well-characterized by monomer occurrences. In the living cell, sequence patterns for realization of relevant signals have to be selective and recognition of the these patterns is usually robust with respect to small variations. Therefore the observed patterns are more complex and show some specific variability. For that reason, in general a collection of longer oligomers, i.e. certain motifs should be more suitable for the modelling of biological signals. The oligo kernel can be used to identify these motifs and provides a tool for analyzing their positional variability. Unlike Markov model based representations, it is not restricted to short oligomers in practice, but may be used for *K*-mers of any length. By means of an effective regularization, characteristic occurrences of longer oligomers may even be found with small data sets, usually encountered in prokaryotic TIS analysis. As already mentioned above, computational cost is even decreasing for longer oligomers.

We made clear that oligo functions are valuable to identify relevant signals in biological sequences. The presented dissection of TIS clearly identifies those areas bearing relevant information. These findings directly influence the design of TIS prediction tools e.g. with respect to the length of up- and downstream regions that have to be analyzed or the selection of discriminative oligomers whose occurrences have to be considered. Attractive features of our approach are its flexibility with respect to oligomer length and position conservation. Both parameters allow an easy adaptation to different biological problems. We conjecture an important role of the oligo kernel in computational biology: In addition to the application presented here, the kernel is well-suited to analyze and model splice sites, transcription factor binding sites or eukaryotic transcription initiation sites. Although we only presented its application to DNA sequences, also sites on protein sequences, like signal peptide cleavage sites are well suited for analysis by means of the oligo kernel. We are currently preparing a web-interface which will allow biologists to perform the analysis, as presented here, on their own sequence data.

## Conclusions

We introduced the oligo kernel for datamining on biological sequences and we showed that it can provide a valuable tool for the identification and analysis of relevant signals. In the case of translation initiation sites (TIS) we could clearly deduce the most discriminative motifs and their positional variation from example sequences. These findings directly influence the design of tools for TIS prediction e.g. with respect to the length of up- and downstream regions that have to be analyzed. Attractive features of our approach are its flexibility with respect to oligomer length and position conservation. By means of these two parameters oligo kernels can easily be adapted to different biological problems. We showed that the position independent spectrum kernel can be viewed as a special case of the oligo kernel and that for the analysis of TIS sequences the incorporation of position information is crucial. In contrast to other position-dependent sequence kernels our approach not only provides learnability of a suitable model but also an easy interpretation of the learnt representation.

## Authors' contributions

PM designed, implemented and tested the oligo kernel and the associated visualization method and he drafted most of the manuscript. RM accounted for biological expertise and substantial parts of the draft. MT contributed in biological expertise and prepared the datasets. BM assisted in coordination and manuscript writing. All authors read and approved the final manuscript.

## Figures and Tables

**Table 1:** Performance of oligo kernel classifiers with oligomer length *K *= 1,...,6. The first line shows the mean classification error, given in percent, on the test sets. The rates are averages over 50 runs on randomly partitioned data. The second line shows the standard deviation of the classification error. The last line shows the mean over the 50 optimal values of *σ *which had been chosen from the set {0.5,0.75,1,1.5,2} for each run to minimize the error on a validation set.

oligomer length	1	2	3	4	5	6
mean (median) error	11.8 (11.8)	9.7 (9.6)	8.9 (8.7)	9.6 (9.5)	12.7 (12.6)	15.0 (15.0)
standard deviation	1.3	1.3	1.4	1.2	1.3	1.2
mean optimal *σ*	0.8	0.8	1.25	1.34	1.24	1.27

**Table 2:** Performance of oligo kernel classifiers on an enlarged data set with four times the number of examples than utilized for the EcoGene-based analysis with results shown in table 1. The oligomer length again varies according to *K *= 1,...,6. The table shows the mean classification error, given in percent, on the test sets. The rates are averages over 20 runs on randomly partitioned data with the same proportions of training, validation and test sets as for the previous results shown in table 1. According to the main paradigm of machine learning we would expect the error to decrease for an increased data set. However, obviously this is not the case, as the error rates are rising up to 6.4 percent, as compared with table 1. Therefore the results indicate that the additional data which have not been experimentally verified, are distributed in a different way than the verified TIS sequences from EcoGene. For that reason we conclude that these additional data should not be used for analysis of TIS, because it cannot be excluded that the distinct distribution is due to erroneous annotation.

oligomer length	1	2	3	4	5	6
mean error	17.3	15.6	15.3	16.0	17.0	18.9

**Table 3:** Comparison of oligo kernels (OK) with inhomogeneous Markov models of order 0 (MM_1_) and order 1 (MM_2_) based on monomer and dimer occurrences, respectively. All higher order Markov models led to a severe breakdown of the performance with an error rising to ≈ 30 percent. The best spectrum kernel (SK) among the position-independent oligo kernels (*σ *→ ∞) with *K *= 1,...,6 is incorporated into the comparison in order to stress the importance of position information. The table shows the mean classification error, given in percent, on the test sets. The rates are averages over 50 runs on randomly partitioned data. The lowest classification error is achieved by the combined oligo kernel OK_1...6 _with simple adding of length 1,...,6 kernels. The combined oligo kernel is closely followed by the best single length trimer kernel OK_3 _which still performs better than the two Markov model based methods. Obviously, the "best" position-independent kernel SP_2_, based on dimer occurrences is performing worst, only slightly better than classification by chance.

method	OK_3_	OK_1...6_	MM_1_	MM_2_	SP_2_
mean (median) error	8.9 (8.7)	8.1 (7.8)	11.4 (11.4)	11.3 (11.4)	44.6 (44.9)
